# Endo and exoglucanases produced by *Penicillium citrinum *isolated from Amazon

**DOI:** 10.1186/1753-6561-8-S4-P179

**Published:** 2014-10-01

**Authors:** Pamella Suely Santa Rosa Pimentel, Anita Lima de Souza, Ana Tana Rosas Nascimento, Edmar Vaz de Andrade, Spartaco Astolfi-Filho, Carlos Gustavo Nunes-Silva

**Affiliations:** 1 Federal University of Amazonas, Manaus, AM, Brazil; 2 Biotech Amazonia LTDA-ME, Manaus, AM, Brazil; 3 Federal University of Amazonas, Biotech Amazonia LTDA-ME, Manaus, AM, Brazil

**Keywords:** Cellulases, Enzymatic production, Penicillium citrinum

## Background

Cellulolytic enzymes (glucohidrolases - EC 3.2.1.-) are biocatalizators highly specific. They act in synergy to hydrolyze β-1,4 bonds between monosaccharide units of D-glucose in the cellulose chain releasing its constituents. Cellulases are categorized according to the place they act in the cellulosic fiber. Endoglucanases start hydrolysis, exoglucanases act in the reduced terminal produced by endoglucanases followed by β-glycosidase which act in the product of exoglucanases catalysis releasing glucose monomers.

Fungi are considered the best cellulolytic enzyme producers due to its natural cellulases that complete saccharification of lignocellulose. Species of *Penicillium *have been reported as excellent producers of cellulolytic enzymes when compared to commercial species and strains [1]. Aiming to contribute to biocatalytic processes and obtention of new sources for cellulolytic enzyme, this work has as objective the production of endoglucanases and exoglucanases from *Penicillium citrinum *isolated from an agro industrial residue in Amazon.

## Methods

Sample was isolated from sugar-cane bagasse in agro industry of Amazonas, by the municipality of Presidente Figueiredo-AM-Brasil, 110 km from Manaus. Molecular identification was made by ITS-18S gene amplification, followed sequence analysis of using software Bioedit Sequence Alignment Editor^® ^and BLAST from NCBI (BLASTn). For the production of cellulolytic enzymes fungi was cultivated during 192h in submerse fermentation, having carboxymethylcellulose (CMC) as carbon source [2]. Enzymatic assays were done in triplicate [3,4] incubating the culture supernatant in presence of substrates CMC for endoglucanases and avicel (microcrystalline cellulose) for exoglucanases. Total secreted protein was quantified by using BCA kit (Thermo Scientific^®^). Enzymatic activity was calculated according to international unities- IU (1 IU is equal to 1 µmol of released product) and, the values were presented in U/mg. As experimental control all the assays were also done with a commercial strain of *Trichoderma reesei *(QM 9414).

## Results and conclusions

Fungi specie was confirmed as *Penicillium citrinum*, having 100% identity and similarity in BLASTn alignment. Regarding to endoglucanase production was observed two peaks, one with 72 h and with 144 h of cultivation, and enzyme activity of 9.67 U/mg and 8.32 U/mg, respectively. This results were comparable to those obtained for *T. reesei *(QM 9414), which was observed activity equal to 12.50 and 14.37 U/mg respectively. Exoglucanase activity was higher in *P. citrinum *(0.64 U/mg, 96h) than that in *T. reesei *(0.43 U/mg, 168h), as indicated by Singh and co-authors [5]. Production profile of endoglucanases and exoglucanases of *P. citrinum *showed to be highly synergic since the exo activity increases gradually and its peak is related to endo activity decay. Therefore *P. citrinum *presents a good potential as alternative to celulolytic enzyme production.
